# Advances in Enzymatic Production of Prebiotic Oligosaccharides from Agro-Industrial Waste: A Critical Review and Industrial Framework

**DOI:** 10.3390/foods15122156

**Published:** 2026-06-15

**Authors:** Slim Smaoui

**Affiliations:** Laboratory of Microbial and Enzymes Biotechnology and Biomolecules (LMEBB), Centre of Biotechnology of Sfax (CBS), University of Sfax-Tunisia, Road of Sidi Mansour Km 6, P.O. Box 1177, Sfax 3018, Tunisia; slim.smaoui@yahoo.fr or slim.smaoui@cbs.rnrt.tn

**Keywords:** prebiotic oligosaccharides, agro-industrial waste, microbial fermentation, food application

## Abstract

Agro-industrial wastes are abundant, low-cost feedstocks used for the sustainable production of prebiotic oligosaccharides via enzymatic methods. This review summarizes recent advances, with particular emphasis on studies published after 2020, in the enzymatic valorization of these by-products, highlighting pretreatment strategies, enzyme classifications, and reaction conditions for converting complex substrates into functional prebiotic ingredients. In parallel, pioneering studies are recognized as foundational work, as they offer key mechanistic insights and continue to play a key role in supporting the interpretation of recent advances in the field. The functionality of the resulting prebiotic oligosaccharides, assessed through various in vitro models, is discussed, with emphasis on their impact on the technological performance of different food matrices. Evidence from in vitro and human studies further illustrates the biological activity and added value of waste-derived prebiotic oligosaccharides. While enzymatic technologies demonstrate high efficiency and selectivity, the full potential of many agro-industrial wastes for bioconversion remains underexplored. Optimizing enzymatic processes and systematically assessing functionality are essential in order to fully harness these resources, supporting the development of innovative, value-added food products within a circular bioeconomy. This review provides an integrated platform linking prebiotic oligosaccharide production, functionality, and food applications, promoting the valorization of agro-industrial waste into high-value prebiotic ingredients.

## 1. Introduction

Lately, there has been a greater awareness of the agro-waste potential as a green resource for exploiting high-value products [1]. In this line, converting agro-waste conforms to circular economy principles, which efficiently upgrade waste into marketable products [2]. To generate agro-waste for resourceful utilization in food, biomedical, and cosmetic industries, numerous value-addition approaches have been industrialized. Agro-industrial wastes, such as sugarcane bagasse, wheat bran, fruit peels, and corncobs, characterize rich, cheap, and renewable raw materials used to produce value-added compounds [2]. Advanced technologies have qualified the conversion of waste materials into valuable products and bio-based compounds [3,4]. Since food waste often comprises high levels of carbohydrates and fibers, it is open to enzymatic processing. These fractions could constitute a precursor for the enzyme-mediated synthesis of prebiotic oligosaccharides, and can be used for the production of prebiotics [1,5].

By definition, prebiotics, grouped into three chief clusters, (i) polyols, (ii) oligosaccharides and (iii) fibers, are non-digestible food components, remain unhydrolyzed by gastric conditions and human digestive enzymes, remain resilient to digestion until they reach the colon, and are differentially fermented by gut microbiota [6,7]. This fermentation helps the development and activity of beneficial bacteria (e.g., *Bifidobacteria* and *Lactobacilli*), limits the growth of pathogenic microorganisms such as *Salmonella* spp. and *Escherichia coli*, and leads to the production of health-promoting metabolites and a general enhancement of host health [1]. Prebiotics can be synthesized by the enzymatic hydrolysis of polymers, as oligosaccharides synthesize through transglycosylation reactions [8]. In this way, enzymatic synthesis has emerged as an effective alternative to chemical glycosylation for prebiotic oligosaccharide production. Attributed to their high selectivity and specificity, enzymes simplify the synthesis of larger and tailor-made oligomers under eco-friendly conditions, with fewer reaction phases, condensed waste generation, and advanced yields compared with conventional chemical methods. Furthermore, the extensive availability of agro-industrial wastes and their contribution to environmental pollution underscore the need to prioritize their valorization within the circular bioeconomy framework. Because of their high specificity, low-severity conditions, and reduced environmental footprint compared with conventional hydrolysis methods, enzyme-based processes have attracted important attention. These sustainable approaches could manage the depolymerization of complex polysaccharides into functional oligosaccharides with distinct degrees of polymerization (DP), which is central for adapting prebiotic activity and technological performance characteristics.

Although advantageous, existing industrial conversion technologies have faced numerous serious limitations that limit large-scale and sustainable production. Conventional acid hydrolysis processes frequently suffer from reduced selectivity, triggering extreme depolymerization, the development of inhibitory by-products, and significant energy demand, which together decrease process efficiency and product integrity. Similarly, physical pretreatment methods require severe working conditions that impair the regulation of structural stability, increase operational costs and constrain scalability. While enzymatic skills characterize a more workable alternative, their industrial deployment is remaining controlled by enzyme cost, structural rigidity, and restraining substrate accessibility.

Interestingly, prebiotic oligosaccharides, like fructooligosaccharides (FOSs), xylooligosaccharides (XOSs), galactooligosaccharides (GOSs) and isomaltooligosaccharides (IMOSs), are low-molecular-weight carbohydrates that consist of monosaccharide residues united by glycosidic bonds, and the number of residues ranges from two to ten units [9]. Naturally, they occur in vegetables and fruits, and can be produced by the enzymes expressed in bacteria, yeast and fungi [10]. Regarding prebiotic oligosaccharide functionality, the structure, the nature of glycosidic linkages, the solubility, the fermentability, the viscosity and DP are the main determining factors. Similarly, prebiotic oligosaccharides mitigate the hazard of numerous disorders, including cardiovascular diseases, inflammation, obesity, diabetes, cancer, and infections [8,11]. Prebiotic oligosaccharides have been pronounced as valuable constituents of the food industry, since they can usually form gels with high water-holding capacity, and improve antimicrobial and antioxidant properties. In addition, prebiotic oligosaccharides are considered functional ingredients and have been undeniably connected to human health [12]. Additionally, the technological properties of prebiotic oligosaccharides make them very valuable ingredients for the food industry.

In response to the increasing need for healthier food products fortified with prebiotic oligosaccharides, this work discusses the current advances in the production of prebiotics from agro-industrial wastes by means of enzymatic technologies, and examines the applications of these emerging technologies in prebiotic foods, in addition to their significance to the food industry.

## 2. Advanced Mechanistic Evidence for Prebiotic–Microbiota Interactions

Prebiotics, special food compounds that sustain helpful gut microorganisms and support human health, are not digested in the small intestine or the stomach [13]. Their health benefits are resolute not only due to the development of specific bacteria, but also due to the connections among these microorganisms and their generation of beneficial metabolites [1].

### 2.1. Fermentation of Prebiotics and Short-Chain Fatty Acid (SCFA) Production

Throughout fermentation, gut bacteria hydrolyze complex carbohydrates into smaller molecules and generate short-chain fatty acids (SCFAs) [14]). These later include acetate, propionate, and butyrate, and play complementary roles in host and microbial metabolism [13]. Acetate promotes lipid metabolism and acts as an energy substrate for other microorganisms, promoting cross-feeding interactions within the gut ecosystem [15]. Propionate, mostly involved in glucose metabolism and in regulating cholesterol synthesis, aids in systemic metabolic balance [16]. Butyrate provides a primary energy source for colon cells and plays a key role in preserving intestinal barrier integrity and overall gut health [17].

### 2.2. Microbial Cross-Feeding and Gut Ecosystem Stability

In the gut, microorganisms can create a supportive network to exchange metabolites through cross-feeding interactions. As an illustration, *Bifidobacterium* and *Lactobacillus* can produce lactate and acetate, which are then exploited by butyrate-producing bacteria such as *Faecalibacterium prausnitzii* and *Roseburia* spp., thereby assisting in the creation of a stable and balanced microbial ecosystem [18].

### 2.3. Molecular Signaling and Health Effects Beyond the Gut

During prebiotic fermentation, metabolites could serve as signing molecules able to induce host cellular functions [19]. Amongst them, butyrate has attracted particular attention because it can control gene expression through epigenetic mechanisms. SCFAs are involved in numerous signaling pathways which are linked to inflammation, immunity, and energy metabolism [17]. These pathways help order immune responses, decrease oxidative stress, and enhance metabolic balance [17].

### 2.4. Prebiotic Structural Features and Their Influence on SCFA Profiles

A greater insight into the connection between prebiotic structure and characteristic SCFA profiles is vital to support mechanistic insights into gut fermentation. Prebiotics, like XOSs, FOSs, IMOs and GOSs, diverge in terms of their monomer composition, glycosidic linkages, and DP, which directly modulate their selective exploitation by gut microbiota and subsequent metabolic outputs [15]. As an illustration, FOSs and GOSs, which are characteristically quickly fermented by *Lactobacillus* and *Bifidobacterium* species, are linked to the increase in the production of lactate and acetate production, which can then feed cross-feeding pathways resulting in butyrate development [16]. In contrast, because of the β-1,4-xylosidic pillar, XOSs typically encourage more diverse microbial fermentation, encompassing *Bifidobacterium* and certain butyrogenic bacteria, contributing to a more balanced SCFA profile characterized by acetate and butyrate [16]. IMOS, being further slowly hydrolyzed, normally displays slower fermentation kinetics, which may control a more sustained SCFA release, slowing rapid acidification compared to the readily utilized oligosaccharides by gut microorganisms [9]. Generally, these structural changes impact not only on microbial selection but also fermentation rate, cross-feeding interactions, and SCFA delivery. Connecting specific prebiotic structures and SCFA profiles would expand mechanistic insights and lead to the design of prebiotics with desired biological responses.

## 3. Microbial Enzyme-Mediated Production of Prebiotic Oligosaccharides

### 3.1. Production of Fructooligosaccharides (FOSs) via Fructosyltransferase and β-Fructofuranosidases Activities

Fructosyltransferases (FTases) are the enzymes that are responsible for FOSs and catalyze the transfructosylation of a fructose unit to a sucrose or FOS molecule, which leads to augmentation in fructans’ DP [20,21]. Depending on the microorganism and culture media, FTase characteristics, such as structures, MW (molecular weight), substrate specificity, glycosylation degree aptitude, and chemical susceptibility, could be modified [22].

In relation to FTases biosynthesis, despite taxon-specific variability, the FOSs produced by bacteria, fungi, and yeasts contain β(2→6) (levan-type) or β(2→1) (inulin-type) linkages [23]. FTases fit to the enzyme family of glycosyl hydrolases, GH32 and GH68, and both are classified within the GH-J clan [24]. The GH32 family comprises various kinds of enzymes like FTases (1-SST, 1-FFT, 6-SST, 6-SFT, 6G-FFT, levansucrases), fructan exohydrolases (FEH: 1-FEH (EC 3.2.1.153), 6-FEH (EC 3.2.1.154), β-fructofuranosidases (FFases), vacuolar invertases (V-INV), cell wall invertases (CW-INV), inulinases, and levanases [25]. The GH68 family contains bacterial inulosucrases, levansucrases and FFases. FTase shifts fructosyl units from sucrose to other sucrose or GFn (glucose-terminated fructooligosaccharides (GFn, n = 2 ~ 8) molecules, whereas FFase displays transfructosylation activity at high sucrose concentrations and hydrolytic activity at low sucrose concentrations [26].

FTases have been described in numerous filamentous fungi, including *Aspergillus* (*A. niger* and *A. oryzae*), *Aureobasidium* (*Aureobasidium pullulans*), *Fusarium* (*F. oxysporum*), *Neurospora* (*N. crassa*), *Penicillium* and *Rhizophus* [20].

Certain fungi, for example *Aspergillus terreus* or *Aspergillus japonicus*, develop FFases and FTases activities [27] which are associated with the sucrose concentration in the medium. For example, at high sucrose concentrations, FFases and FTases indicated transfructosylating activity, while at low sucrose levels, the FFases demonstrated hydrolytic effects over fructans [28]. In *A. niger*, FTase displayed only transfructosylation activity, while its FFase did not reveal fructosyltransferase properties [29]. Interestingly, *A. niger* AS0023 could produce FFases and FTases, and *ureobasidium pullulans* are judged a source of FTases and FFases [20]. On the other hand, the utilization of sucrose-rich industrial by-products can be an inexpensive and gainful alternative when generating FOSs. To produce FOSs by FTase from *Aspergillus flavus* NFCCI 2364, Ganaie et al. [30] employed solid-state fermentation to explore sixteen different agro-wastes. Under non-optimized parameters, sugarcane bagasse was a suitable substrate with a yield equal to 36% (*w*/*w*). After process optimization, higher levels of FOSs were generated (73.42%, *w*/*w*), composed of 1-kestose, nystose and 1F-fructofuranosylnystose at 46.3, 22, and 21%, respectively. Under solid-state fermentation, Muñiz-Márquez et al. [31] produced FOSs with FTase from *Aspergillus oryzae* DIA-MF. In this study the aguamiel of maguey was employed as a culture medium; a yield of 0.30% (*w*/*w*) of FOSs was attained, with a productivity equal to 97 mg/(L·min). Through solid support in solid-state fermentation, Mussatto and Teixeira [32] reported that FOS production employing coffee silverskin was more suitable than synthetic fiber. Coffee silverskin provides appropriate nutrients that tolerate microbial growth, attaining findings matching those of nutrient-enriched media. Under these conditions, raised FFase activity was perceived: FOS yield = 0.70% (*w*/*w*) with a productivity of 8 g/(L·h). These authors reported that SSF with coffee silverskin and *A. japonicus* can be measured as an exciting tool for producing FOSs and FFase at the industrial scale. Likewise, Guimarães et al. [32] used agro-industrial by-products as carbon sources, and the optimum temperature/pH was 60 °C/4.5, respectively. The purified enzyme presented respectable thermal stability and had a half-life of 53 min at 60 °C. At 1 mM, FFases activity was marginally activated by Cu^2+^, Mn^2+^, Mg^2+^, and Na^+^. In addition, the enzyme decomposed inulin (1.74 mM), sucrose (5.78 mM), and raffinose (5.74 mM), displaying their respective Kd values. In addition, as highlighted by Zhang et al. [33], *Aureobasidium melanogenum* has a strong ability to transform molasses into FOSs with high FFase activity. The CREA gene, which codes for a glucose repressor in the FFases–producing *Aureobasidium melanogenum* 33, was disturbed, thereby alleviating glucose repression in mutant D28. D28 attained ~2.1 × 10^3^ U/mL, compared to <0.6 × 10^3^ U/mL in the parent and recombinant strains. With D28 cells, cane molasses at 350 g/L sugars were quickly transformed into FOSs, attaining a yield of 0.58 g/g in 4 h. In addition, date by-products have been positively exploited with immobilized enzymes from *Aspergillus awamori*, attaining effective FOS production [34]. In this study, the immobilized FFase transformed 84% of sucrose into 123 g/L of FOSs, with a yield of 53% and a productivity of 18 g/(h·100 g). Table 1 highlights illustrative systems for FOS production, counting the substrates used, the enzymes or microorganisms involved the main production conditions, and the key results in terms of yield and productivity.

### 3.2. Microbial Xylanases in Xylooligosaccharide (XOS) Production

Generated from xylan, the xylooligosaccharides (XOS) were the important constituent of lignocellulosic biomass, covered by cellulose, hemicellulose and lignin at 30–50%, 20–40%, 15–25%, respectively [34,37]. For XOS production, the application of lignocellulosic biomass has been exploited and viewed as a major source offering a stimulating key to fulfill growing XOS requests with a negligible ecological footprint [42,43].

In this way, in order to produce XOS, enzymatic hydrolysis is the best biological approach. Via monosaccharide dehydration, xylan enzymatic hydrolysis is preferred over the chemical method since it limits the generation of compounds like furfural and hydroxymethylfurfural (HMF) [44]. In the xylan skeleton, xylanases could cleave the β-1,4 linkages and contribute to a complex hemicellulose into smaller sugars like xylooligosaccharides and xylose [45]. In such a case, complete hydrolysis typically requires a whole xylanolytic enzyme system, counting especially endoxylanases (E.C.3.2.1.8) and β-xylosidases (E.C.3.2.1.37), which are the two key enzymes that cleave the xylan chain. β-xylosidases catalyze these xylooligomers, liberating xylose, while endo-xylanases affect the homopolymeric backbone of 1,4-linked β-d-xylopyranose, producing xylooligomers [46,47]. Owing to its enzymatic profile, each microorganism exhibits a single XOS utilization pattern. For instance, XOS-containing uronic acids are processed by a few human fecal *Bifidobacteria* [13]. It has been recognized that *Lactobacillus brevis* strains convert only linear XOSs while *Bifidobacterium adolescentis* can catabolize both arabino-XOSs and linear XOSs [12,43]. On account of their high selectivity’s towards xylans, the fungal and bacterial xylanases of glycoside hydrolase (GH), the GH10 and GH11 families, with an endo-1,4-β-xylanase activity, are widely employed for XOS synthesis [48,49].

To date, the GH5 family incorporates 54 sub-families, and the members exhibited multiple activities, such as endo-β-1,4-glucanases (EC 3.2.1.4), endo-β-1,4-mannanases (EC 3.2.1.78), and endo-1,4-β-xylanase activity [50]. The generation of GH5 xylanases by *Bacillus* subtilis 168 (XynC), and *Erwinia chrysanthemi* D1 (XynA) has been described by St. John et al. [51] and Carvalho et al. [52], respectively, and it has been revealed that these enzymes degrade glucuronoxylan to ramified xylooligomers. Gallardo et al. [53] stated that a distinct GH5 xylanase from *Bacillus* sp. strain BP-7 (Xyn5B) had the capacity to degrade linear XOSs with higher DP, and the production of methylglucuronic compounds and xylooligomers, and, on the other hand, the synthesis of GH5 xylanase by Aeromonas caviae ME-l (xynD), have also been noted [54]. The GH7 enzyme from Trichoderma reesei is reviewed as a non-specific endo-β-1,4 glucanase since it has the capability to degrade β-glucan, xylan, and cellulose [55]. From *Bacillus* polymyxa, the GH43 enzyme demonstrated both α-l-arabinofuranosidase and xylanase activities [56]. In the GH8 family, produced by *Pseudoalteromonas haloplanktis* TAH3a, this enzyme exhibited greater activity toward long-chain XOSs with the production of xylotetraose and xylotriose [57]. Regarding the GH10 family, which consists of endo-1,4-β-xylanases and endo-1,3-β-xylanases, *Streptomyces lividans* [58], *Thermoascus aurantiacus* [59], Cellvibrio japonicus [60], and *Geobacillus stearothermophilus* [61] constitute some examples of strains biosynthesizing the xylanases. Concerning GH11, endo-1,4-β-xylanase and exo-1,4-β-xylosidase activities are found in this family. It has been documented in numerous investigations that the major liberated products released after the hydrolysis are xylotriose and xylobiose [62]. In comparison with GH10 xylanases, GH11is characterized by high pI values and a low molecular mass. *B*. *subtilis* B230, *Streptomyces* sp. S38, *Aureobasidium pullulans* NRRL Y-2311–1, *Aspergillus kawachii*, *Penicilium funiculosum*, and *Thermomyces lanuginosus* are well-known strains that are producers of GH11 xylanases [50].

The organic waste bioconversion, viz. lignocellulose wastes from agro-industrial processes, is commonly achieved by the use of hydrolytic enzymes called hydrolases. As an illustration, Ávila et al. [63] produced XOSs from xylan derived from coffee husk (CH) and sugarcane straw (SS) by employing an endo-xylanase (GH11), α-L-arabinofuranosidase (GH51) and feruloyl esterase (CE1). The effective enzymatic blend attained a high total XOS at 10.23 and 8.45 g/L of xylan for SS and CH, respectively. In addition, these authors reported that the tested *Bifidobacterium* and *Lactobacillus* strains were able to exploit XOS derived from agricultural wastes and revealed good growth, associated with a great prebiotic capacity and good antioxidant potential. From *Bacillus mojavensis* A21, an alkaline xylanase employed the corncob xylan for xylotriose and xylobiose liberation [64]. After xylan decomposition, the xylanase of *Bacillus aerophilus* KGJ2 presented a performance in XOS synthesis, like xylose, xylotriose, and xylobiose [65]. From xylan isolated from raw corncobs, a xylanase gene PbXyn10A from *Paenibacillus barengoltaii* showed an XOS yield equal to 75% [66]. The hydrolysis of xylan using xylanase from *Pichia stipitis* produced 2% XOS, involving xylotriose, xylobiose, and xylotetraose at 49%, 29% and 14%, respectively [67]. Bhardwaj et al. [68] confirmed that xylanase isolated from *Aspergillus oryzae* LC1 presented a potential to generate xylotetraose, xylobiose, and xylotriose. Employing beechwood xylan, Gautério et al. [69] assessed the XOS production at 50 °C, pH 5.5, and 50 rpm/24 h with xylanase from *Clostridium* BOH3 attached to calcium alginate and silica gels. Over seven cycles, the entrapped enzyme preserved its functionality (at 65%), and xylotriose and xylobiose were generated. Recently, from sugarcane bagasse, Kathiresan et al. [70] produced XOSs by enzymatic hydrolysis on alkali-extracted xylan. In 20% NaOH treatment, a maximum yield at 86% was noticed, and the best conditions for XOS production were identified as pH 4.75, 45 °C, and enzyme 4 U/mL, at 16 h. In addition, *L. plantarum* and *L. fermentum* showed the determined prebiotic index (PI) for XOSs from xylan at 1.92 and 1.61, respectively. Qiu et al. [71] purified a heat-resistant xylanase isolated from *Thermomyces dupontii* J22 with an activity equal to 770 U/mL. The crude enzyme displayed the maximum activity for xylanase at 80 °C, pH 6. Derived from hydrothermal treatment of corncob biomass extracts, the studied enzyme was active in producing XOS, and could produce xylotriose and xylobiose at 75% of the total composition. Table 2 shows the recent studies describing XOS production processes, and the source, microorganism, and enzyme, and production conditions.

### 3.3. Microbial Enzyme-Based Production of Isomaltooligosaccharides (IMOs)

Isomaltooligosaccharides (IMOs), oligomers of glucose with α (1–6) bonds, and occasionally α (1–2), α (1–3) or α (1–4) linkages, consist of diverse oligomers, *viz.* isomaltose, isomaltotriose, isomaltotetraose, isomaltopentaose, kojibiose, nigerose, panose, and other structurally intricate branched oligosaccharides [80,81]. By the biocatalytic conversion of α-amylase, β-amylase, α-β glucosidase, dextransucrase, or pullulanase, IMOs are produced from starch, forming maltose and maltotriose, and are succeeded by transglycosylation activity by α-transglucosidase [82]. For transglycosylation mechanisms, glycosyltransferase (EC. 2.4) and GH (EC. 3.2), which comprise GH31, GH13, GH70, and GH57, are used and have been employed as catalytic agents [83]. Using conventional starch hydrolysis tools, the IMO composition was controlled by substrate specificity, hydrolytic enzyme and the reaction condition (temperature and pH). In this way, MOS within a DP of 2–10 offer a platform for the high-yield synthesis of desired saccharides. On the other hand, the commercial production of MOS involves two steps that include starch liquefaction and saccharification, leading to purifications [82].

To generate IMOs, the GH31 (α-glucosidases (EC 3.2.1.20)) with transglycosylation activity was employed, and the maltose was employed as the substrate. The α-glucosidases from *Aspergillus niger* (ANG), *Aspergillus oryzae* (AOG) and *Schwanniomyces occidentalis* (SOG) produce panose and isomaltose as the principal transglycosylation compounds [83]. An α-glucosidase from *Xanthophyllomyces dendrorhous* mainly synthesizes panose [84], whereas the enzyme from *Acremonium implicatum* mainly leads to 4-α-nigerosyl glucose [85]. An alternative GH31 member connected to IMO production is the 6-α-glucosyltransferase from *Paenibacillus* sp. 598K [86]. From the non-reducing end of starch, this enzyme could liberate glucose and extend α-1,6-linked glucooligosaccharides, resulting in a linear IMO with a DP between 4 and 10 [87].

The family GH13 enzymes cover maltogenic amylases (EC 3.2.1.133), cyclomaltodextrinases (EC 3.2.1.54) and neopullulanases (EC 3.2.1.135) [88]. In the maltogenic amylase OPMA-N from *Bacillus* sp., which is capable of utilizing starch as a substrate to produce isomaltotetraose and isomaltotriose, the largely preserved Trp358 residue has been closely connected to transglycosylation activity [89]. In the maltogenic amylase from *Thermus* sp. (ThMA), the mutation of Glu332 to histidine substantially limited α-1,6 transglycosylation without altering hydrolytic activity. The GH70 family, linked to IMO production, consists of numerous kinds of transferase enzymes, comprising alternansucrases, reuteransucrases, 4,3-α-glucanotransferases, 4,6-α-glucanotransferases, dextransucrases, mutansucrases, and α-1,2- dextransucrases [35]. The GH70 glucansucrases utilize sucrose instead of starch and transfer glucosyl units to suitable acceptors via α-1,2, α-1,3, α-1,4, or α-1,6 linkages [90]. Glucansucrases are mainly present in Gram-positive lactic acid bacteria (*Weissella*, *Leuconostoc*, *Lactobacillus*, and *Streptococcus*) and in Gram-negative bacteria (*Exiguobacterium*, *Azotobacter*, and *Paenibacillus*) [91]. From *Azotobacter chroococcum* NCIMB 8003, Gangoiti et al. [92] isolated a thermostable α-4,6-glucanotransferase with a maximum of activity (60 °C/pH 6.5). These authors also described an α-4,3-glucanotransferase from *Lactobacillus fermentum* NCC 2970 [92]. GH57 branching enzymes (EC 2.4.1.18) convert α-1,6 branch formation by hydrolyzing α-1,4 bonds in amylose and shifting the liberated oligosaccharides to a glucan chain via an α-1,6 linkage [91]. The branching enzyme from *Thermococcus kodakaraensis* KOD1 has a molecular structure integrated into central catalytic domains A and B, a C-terminal domain, and an additional α-helical subdomain [93]. The IMOs have been produced from a wide diversity of natural by-product sources comprising starch, maltose and maltodextrin (Table 3). As an illustration, from an inexpensive sweet potato starch (SPS), Duong Hong et al. [94] established a simple two-step process to generate IMOs. Liquefaction with Spezyme Xtra, a thermostable α-amylase enzyme preparation produced by a genetically modified strain of *Bacillus stearothermophilus*, produced 74% (G1–G10) and 50% (G2–G6). Under optimal conditions, subsequent SST (β-amylase, pullulanase, α-transglucosidase) generated 70 g/L of IMOs. From potato peel starch, Maurya et al. [95] applied liquefaction, saccharification, and transglucosylation to generate IMOs, and an α-transglucosidase from *Aspergillus niger* GH31 was cloned and recombinantly expressed in *E. coli* BL21, then purified, and fixed onto magnetic nanoparticles, exhibiting activity until 62% after five cycles. Optimized transglucosylation was attained at these conditions (pH 5.5, 45 °C, 6.9 U/g and 9 h), producing 70 g/L of IMOs. For IMO production, Basu et al. [96] engineered single-step concurrent saccharification and transglucosylation (SST) from starch. α-Glucosidases (1.05 U/5 mL and Fungamyl 800 L (0.06 U/5 mL) were optimized using the Nelder–Mead algorithm. Under optimal conditions, SST produced 96 g/L IMOs in 12 h, and the profile product comprised panose and isomaltose at 39.40 and 13.83 g/L, respectively. According to these authors, the developed SST was a well-organized and workable strategy for IMO production since the yields were 92 and 85 g/L of IMO from potato waste and broken rice, respectively. Later, Tiangpook et al. [97] investigated the production of long-chain IMOs by the direct fermentation of maltose using *B. subtilis* AP-1. Via maltose fermentation, this strain could produce long-chain IMOs (DP 2–14), forming 37 g/L at 36 h. The produced IMOs presented robust prebiotic properties and no cytotoxicity (≤5 mg/mL). When *Zalaria* sp. Him3 was incubated at 150 g/L maltose for 48 h, the IMO production was realized using α-glucosidase, with a maximum of 138 g/L after 12 h from 250 g/L maltose (which corresponds to 98% conversion within 72 h) [98]. In addition, panose, isomaltotriose and isomaltose are the major found products.

### 3.4. Microbial Enzyme-Based Production of Galactooligosaccharides (GOSs)

Galactooligosaccharides (GOSs), generated by the transgalactosylation of lactose by the β-galactosidase, were tested in lactose-rich substrates such as milk and milk whey [102]. The reaction, resulting in GOS synthesis, can be classified into three main steps: (i) hydrolysis, (ii) the production of a galactosyl-enzyme intermediate, and (iii) GOS synthesis [102]. For lactose hydrolysis, extracted β-galactosidases from bacterial genera, viz. *Arthrobacter*, *Lactobacillus*, *Bifidobacterium*, *Enterobacter*, *Pyrococcus*, *Bacillus*, *Escherichia coli Propionibacterium*, *Streptococcus*, and *Beijerinckia*, have been lengthily employed for lactose conversion (Table 4). In a corn steep liquor–whey medium complemented with soybean oligosaccharides as the principal carbon source, Han et al. [103] generated α- and β-galactosidases by *Bifidobacterium longum* subsp. longum RD47. By Response Surface Methodology (RSM), an optimized whey–tryptone medium allowed the psychrotrophic bacterium *Enterobacter ludwigii* to yield 35 U/mL of cold-active β-galactosidase, indicating a 3.6-fold increase compared with the unoptimized medium [104]. Similarly, a batch fermentation of *Lactobacillus leichmannii* 313 was optimized by RSM, and produced 23.5 U/mg of β-galactosidase after 12 h at 30–55 °C and pH 7.5 [105].

On the other hand, *Kluyveromyces lactis*, *K. marxianus*, *K. fragilis*, *Candida kefyr*, and *Saccharomyces cerevisae* represent the yeast sources, while *Aspergillus oryzae*, *A. niger*, *A. aculeatus*, *A. carbonarius*, *Penicillium expansum*, *P. chrysogenum*, and *Paecilomyces aerugineus*, are some of the fungal sources identified as producers of β-galactosidase [106]. Nakagawa et al. [107] screened a psychrophilic yeast, *Guehomyces pullulans*, for the production of extracellular and acidic β-galactosidase. The strain was cultivated on lactose as the sole carbon source below 5 °C and at pH 4.0, leading to 1.2 U/mg protein and representing the possibility for low-temperature enzyme production suitable for whey and milk hydrolysis. *Kluyveromyces marxianus* NCIM 3465 cells were chemically treated with 50% (*v*/*v*) ethanol to overcome lactose transport limitations. These authors observed an 89% hydrolysis of skim milk lactose with a maximum β-galactosidase activity of 1.54 IU/mg [108]. During growth in a synthetic medium, a novel Aspergillus species *A. lacticoffeatus* efficiently generated 450 U/mL β-galactosidase. It was successfully able to catalyze the synthesis of prebiotics such as lactulose (2.5 g/L) and GOSs at 2.5 and 6.3 g/L, respectively [109]. A novel β-D-galactosidase-producing strain, *Teratosphaeria acidotherma* AIU BGA-1, was isolated from an acidic hot spring in Japan [110]. The microorganism, cultivated at pH 2.0–4.0, could produce four intracellular β-D-galactosidases with diverse pH optima, including two novel extremely acidophilic enzymes (optimal pH 1.0 and 3.0–3.5) and one novel alkalophilic enzyme (optimal pH 8.0), stressing their potential for low-lactose dairy applications.

Additionally, recombinant microbial GOS production systems represent efficient platforms for the industrial sector. In this context, β-galactosidases have been planned using diverse recombinant and synthetic biology methods to improve their exploration in industrial biocatalysts. In this line, some thermostable β-galactosidases were isolated from recombinant *Geobacillus stearothermophilus*, *Pyrococcus furiosus Sulfolobus solfataricus*, and *Thermotoga maritima*, etc., and presented improved purification efficiency and elevated activity [111]. During incessant fermentation in an airlift bioreactor covering lactose at 50 g/L as the substrate, Domingues et al. [112] synthesized heterologous β-galactosidase by a recombination of the *Saccharomyces cerevisiae* strain with the β-galactosidase gene of *A. niger*. In comparison with batch culture, a productivity of 6.2 × 10^5^ U/h was attained, representing 11-fold growth [112]. Plantz et al. [113] revealed that the product yield of recombinant β-galactosidase can be impacted by the levels of trace metals as detected in recombinant *Pichia pastoris* grown in a distinct medium with Mg and Zn ions as support for β-galactosidase production. From *Alicyclobacillus acidocaldarius*, Di Lauro et al. [114] isolated thermostable, at 65 °C, and recombinant β-galactosidase. A thermostable β-galactosidase gene from *Bacillus stearothermophilus* was successfully cloned and expressed in *Bacillus subtilis* WB600, yielding a recombinant enzyme with optimal activity at pH 7.0 and 70 °C [115,116].

**Table 4:** Examples of culture conditions for β-galactosidase production by bacterial and fungal microorganisms.

Source	Microorganism	Enzyme	Production Conditions	Main Results	References
Lactose solution	*Bifidobacterium longum* BCRC 15708	β-galactosidase	40% (*w*/*v*) lactose; 45 °C; pH 6.8	GOS: 32.5% yield (*w*/*w*), mainly trisaccharides; lactose conversion 59.4%	[117]
Lactose solution	*Penicillium expansum* F3	Immobilized β-galactosidase in Ca-alginate	380 g/L lactose; pH 5.4; 50 °C; repeated batches	GOS purity 28.7% (*w*/*w*); non-monosaccharide GOS purity > 37% (*S. cerevisiae*) and up to 97% (*K. lactis*)	[118]
Lactose solution	*Thermotoga maritima* (BglA, β-glucosidase)	Thermostable β-glucosidase (GOS-forming)	At 570 g/L lactose; optimized for transglycosylation	Very high GOS yield 72.1% (wt%) from lactose; mainly β(1 → 3) and β(1 → 6) linkages	[119]
Gum arabic	*Kluyveromyces* lactis (whole cells)	Intracellular β-galactosidase	Galactose from gum arabic hydrolysate; 35 °C/pH7.6	GOS = 45%; mixture contained only GOS and unreacted galactose, cells reusable ≥ 30 batches	[120]
Lactose/lactulose	*Lactobacillus delbrueckii* subsp. bulgaricus CRL450	β-galactosidase	Lactose or lactulose as substrates; optimized lab conditions	From lactose: 41.3% GOS; products mainly β(1 → 6) and β(1 → 3)	[121]
Lactose solution	*Pseudomonas tritici* SWRI145	Novel β-galactosidase (0.4 mg/mL)	300 g/L lactose; 40 °C; pH 8.0	Produced 134.3 g/L GOS (44.8% yield) with 80% lactose conversion; DP2–4 GOS	[122]

## 4. Structure–Function Basis of Prebiotic Oligosaccharide Formation

Via a shared catalytic mechanism connecting the development of a covalent glycosyl–enzyme intermediate, retentive glycoside hydrolases induce both hydrolysis and transglycosylation [123,124,125,126,127,128,129,130]. The key transformation rises throughout the second catalytic stage, where the enzyme must choose between water or another sugar molecule as the acceptor [131]. In hydrolysis, water reacts with the intermediate, subsequent to the cleavage of the glycosidic bond and the liberation of smaller sugars [132]. However, in transglycosylation, a sugar acceptor participates with water, letting the enzyme build new glycosidic linkages and create oligosaccharides with prebiotic potential [133]. The reaction promotes hydrolysis or transglycosylation strongly based on both the enzyme structure and substrate chemistry. At the molecular level, the architecture of the dynamic position acts as a conclusive role: hydrophobic or aromatic residues in positive subsites can increase sugar compulsory while restraining water accessibility, thus fluctuating the reaction toward transglycosylation [134]. Similarly, small changes in hydrogen-bonding nets, catalytic water positioning, or loop flexibility can control the energetic dividing between hydrolysis and sugar transfer [135]. Enzymes with supplementary elastic loops or abridged water-channel accessibility frequently show improved transglycosylation/hydrolysis ratios as sugar acceptors are steadied more efficiently than water molecules throughout the transition reaction [136]. Substrate properties are similarly significant. Generally, galactomannan or xylan could promote hydrolysis because their prolonged chains bind across numerous enzyme subsites while water remains freely mobile and abundant [137]. Conversely, small sugars like lactose or sucrose fit capably into a compact −1/+1 binding site, and at high levels they can positively outcompete water, indorsing transglycosylation and prebiotic oligosaccharide synthesis. This clarifies why high donor concentrations often look to slow hydrolysis while increasing oligosaccharide formation. Outstandingly, transglycosylation products often pass because they may later be hydrolyzed again, generating a dynamic kinetic balance between production and degradation [138]. Overall, the divider between hydrolysis and transglycosylation is controlled by an indirect interaction between the architecture of enzyme, water organization, and physical substrate chemistry, which collected promote if the enzyme primarily disruptions glycosidic bonds or produces new prebiotic carbohydrates.

## 5. Impact of Agro-Waste Variability on Enzymatic Consistency

The prebiotics production from agro-industrial and food waste was governed by the consistency of enzymatic hydrolysis, which itself needs a stable feedstock composition. However, waste resources are integrally inconstant because of seasonal, regional, and processing-related factors, creating major challenges in dependable prebiotic production [1]. Differences in carbohydrate, lignocellulosic fiber, protein, ash, moisture, and inhibitor contents can pointedly touch enzyme availability, hydrolysis competence, and the liberation of fermentable oligosaccharides [139,140]. Seasonal changes in fruit and vegetable residues could modify substrate structure and biodegradability, while variations in the group source, storage environments, and co-processing with other wastes alter the pH, salt content, and structural features of the biomass [141]. At the molecular level, variations in lignin, acetyl groups, and other structural blocks could affect hemicellulase and cellulase entree to polysaccharide chains, promoting unreliable sugar release and prebiotic yields [139]. Such variability not only disturbs enzymatic change efficiency but also powers the downstream microbial fermentation employed to generate or augment prebiotic complexes, since microbial development and metabolism are extremely sensitive to carbon/nitrogen balance and hydrolysate composition [142]. Accordingly, at the industrial scale, the utilization of usual feedstock goods can result in a poor process control, changing product quality, and variable techno-economic feasibility. To overcome these restrictions, numerous strategies have been planned, including exhaustive feedstock depiction, substrate classification, the unification and standardization of hydrolysates, and predictive models which are capable of linking biomass composition to enzymatic and fermentation performance [143]. As highlighted by Saini et al. [139] and Chelliah et al. [141], lignocellulosic residues fluctuate by seasonal and geographical factors relating to carbohydrate composition, lignin concentration, and the attendance of inhibitory compounds, requiring substrate-specific enzyme cocktails and process optimization to realize consistent oligosaccharide yields. On the other hand, White et al. [144] evaluated the effects of the growing season, harvest timing, and cultivar on the biomass yield and chemical composition of sugarcane post-harvest residues. This study showed that sugarcane post-harvest residues consistently contained high levels of cellulose and hemicellulose, averaging 36.0% and 31.5%, respectively. These authors reported that the residue biomass yields exceeded 3.5 t/ha/year.

## 6. Downstream Processing of Industrial Enzymatic Prebiotics

The large-scale manufacturing of enzymatic prebiotics, *viz.* GOSs, FOSs, and XOSs, does not end with the enzymatic reaction. In practice, after production, the reaction broth is composite blend that includes not only the oligosaccharides, but also great quantities of residual sugars, enzymes, salts, buffers, and inhibitory or toxic constituents from raw material pretreatment. As a consequence, downstream processing (DSP) develops a critical step that mainly controls the purity, safety, functionality, and economic viability of the final prebiotic ingredient [145]. Frequently, crude FOS or GOS combinations may be equal to 40–60% undesirable mono- and disaccharides such as glucose, fructose, galactose, lactose, or sucrose, which can decrease prebiotic discrimination and the product value if not detached resourcefully. To tackle this challenge, industries characteristically depend on combined purification plans joining numerous separation skills. Membrane processes are frequently employed as the first large-scale purifying stage since they can selectively separate molecules on the basis of charge and size, and work with a relatively low energy demand. Ultrafiltration retains enzymes and greater molecules, permitting enzyme reprocessing and minimizing processing expenses, whereas nanofiltration eliminates slighter sugars and absorbs oligosaccharides, therefore improving the product purity [146]. For developments necessitating great purity, like infant nutrition or pharmaceutical-grade prebiotics, simulated moving bed (SMB) chromatography is regularly used to attain the near-complete separation of oligosaccharides from residual sugars, often exceeding 99% purity [147]. Other methods like EtOH precipitation or solvent-assisted fractionation are also extensively exploited for the reason that they enrich higher-DP oligosaccharides while eliminating smaller sugars and contaminants in a comparatively modest and walkable manner [148]. To exploit recovery, purity, and process efficiency, industrial bioprocesses assimilate these skills into multi-step purification flowsheets uniting membrane cascades, chromatography, and careful precipitation. Overall, downstream recovery is not just a final improving phase but one of the central technological and economic constraints in converting enzymatic synthesis into commercially viable, food-grade prebiotic products.

Techno-economic investigations show that, for relatively simple sugar separations, membrane cascades can reach costs that are comparable to or lower than SMB chromatography at a large scale. However, this economic advantage is highly dependent on separation selectivity, as more challenging systems such as glucose–fructose become costlier with membranes than with SMB. Notwithstanding their advantages, membrane processes face key scalability constraints, such as fouling and flux decline, cleaning requirements, and augmented electricity consumption at the industrial scale. Additional limits like module durability, engineering consistency, and long-term operational stability can further increase operating costs and complicate scale-up. In contrast, chromatographic techniques remain industrially mature and highly selective, particularly for high-purity separations. However, they typically involve higher resin and buffer consumption, significant capital investment, and lower throughput in some applications. Consequently, membrane systems are generally preferred for bulk fractionation, while chromatography is reserved for high-resolution or final polishing steps.

## 7. Food Industry Applications of Prebiotic Oligosaccharides

### 7.1. Fructooligosaccharides (FOSs)

Owing to their functional properties and market significance, FOSs are classified as one of the most important and extensively investigated prebiotics [20]. In this context, the global FOS market is predictable to reach around $1.04 billion by 2027 [11]. Physiochemically, FOSs are non-reducing sugars, showing good stability in the range of 3–7, being stable at temperatures reaching 140 °C, and keeping about 1/3 of the sweetness of sucrose and a low caloric value [11]. They demonstrate nonprecipitating and noncrystallizing properties, rendering them suitable for changing the freezing temperature of food products [149]. Therefore, because of their properties, they do not undergo caramelization or Maillard reactions, and are suitable for incorporation into high-temperature industrially processed foods, such as pasteurized foods [11]. FOSs have numerous applications in the food field. Renuka et al. [150] revealed that FOSs can partly substitute sucrose in fruit juice beverages and maintain both physicochemical stability and sensory quality throughout storage. In mango, orange, and pineapple juices added with 3.4–3.8 g FOS/100 mL, the pH, titratable acidity, total soluble solids, and color continued to be stable at 4 °C and 25 °C. Additionally, sensory assessment established that the beverages maintained acceptable consumer appeal for up to 6 months under refrigerated conditions and 4 months at ambient temperature (25 °C). These outcomes illustrate that FOSs, at concentrations of 3.5–3.8 g/100 mL, can offer prebiotic functionality without compromising product quality or consumer acceptance. Thota et al. [151] examined the incorporation of short-chain fructooligosaccharides (sc-FOSs) in croissants at 25, 50, 75 and 100%. Sc-FOSs could modify water redistribution and dough water-binding features, while having only minor impacts on protein secondary structure. Rheological studies exposed augmented storage and loss moduli, decreased dough deformation, and enhanced elastic recovery, representing the strengthened gluten connections and boosted dough structure. Furthermore, across all formulations, the crust color and crumb porosity were conserved. However, sensory evaluation exhibited that Sc-FOSs at 100% conferred the firmer croissants and decreased the overall acceptability, whereas 25 and 50% preserved an optimal sensory quality and consumer acceptance while increasing nutritional value. Ureta et al. [152] successfully employed FOS/GOS syrups as sucrose replacers in quinoa orange snacks fortified with quinoa flour and grains, collected with orange peel and pulp. The addition of prebiotic syrups decreased the caloric content without unfavorably touching key sensory and physicochemical attributes. Instrumental color, moisture, cohesiveness, and texture were mainly sustained, leading to snacks with a soft texture and good structural integrity. In addition to their favorable technological properties, the snacks displayed considerable polyphenol contents ranging from 80 to 97 mg gallic acid equivalents (GAE)/100, reflecting the contribution of quinoa- and orange-derived bioactive compounds. Liu et al. [153] studied the impact of FOSs (between 0.2 and 0.8%) on the stability of frozen quinoa-based dough. The integration of 0.4% FOS could diminish protein depolymerization by minimizing *α*-helix, and *β*-sheet and disulfide linkages. This fact could preserve the dough matrix integration. The incorporation of 0.4% and 0.6% FOS could improve water mobility via higher hydrogen attachment with proteins, promoting an enhancement of dough’s rheology. After 90 days of storage at freezing temperature, compared to the untreated groups, 0.4% FOS was detected as being linked to enhanced baking functionality, showing a 37% decrease in hardness and a 47% growth in specific volume. FOSs have been estimated as cryoprotectants in gluten-free frozen dough formulations to enhance bread quality and yeast capability throughout freezing [149]. After 7 days at −18 °C, the FOS addition could improve the dough’s development, bread-specific volume, and crumb smoothness. Compared to the control samples, an enhanced formulation covering 70% FOS and 30% hydrolyzed soy protein enhanced the yeast fermentation performance and amplified crumb softness and specific volume by 45% and 41%, respectively.

Undesirably, FOSs negatively affect the rheological behavior of Greek yogurt, creating less consistent, elastic, viscous, and firm products [154]. In the production of Spanish salchichón, FOSs were positively employed as part of fat replacers in a mixture with probiotic strains *Lb. paracasei* and *Lb. rhamnosus*, producing a 30% decrease in fat content [155]. Additionally, FOS/probiotic strains did not affect the sensory features of sausages [155]. FOSs have been assessed as fat alternatives in low-fat and reduced-sodium meat emulsions [156]. FOS addition led to an increase in tenderness and changed the microstructure by helping to form a more porous matrix. The emulsions showed elastic behavior (G′ > G″), while fiber FOS incorporation augmented gelation temperature, signifying reduced myosin gelation. In another study conducted by Salazar et al. [157] studied the addition of sc-FOSs at 2–6% in dry fermented sausages prepared with varying fat contents of 6, 15 and 30% backfat. Throughout ripening, the sc-FOS addition did not alter the physicochemical characteristics or microbial growth, indicating that fermentation and product stability were conserved. Nevertheless, high levels of sc-FOSs could decrease the light-ness (L*) and sausage hardness, contributing to a softer texture and better chewability. For all formulations, sensory assessment exposed good overall acceptance, with the highest consumer preference observed in sausages containing 15% backfat. At 4 and 6% sc-FOSs, the formulated samples provided a desirable balance between texture enhancement, fiber fortification, and sensory features.

### 7.2. Xylooligosaccharides (XOSs)

XOSs displayed numerous helpful characteristics for the food industry in addition to their prebiotic properties. At an acidic pH and high temperatures, XOSs present good stability, have a sweetening power, and can be employed as sweeteners with ∞30% of sucrose’s sweetness [158]. Remarkably, XOSs do not impart an aftertaste and can stop starch retrogradation, thus improving the sensory and nutritional characteristics of foods [11].

Silva et al. [159] confirmed that the orange juice added with XOSs at high-intensity ultrasound (HIUS), at 300–1200 W over 10 min, presented similar characteristics to fresh products. At temperatures ranging between 51 and 88 °C, HIUS did not disturb the chemical stability of XOS. Nevertheless, undesirable impacts of HIUS were observed on the stability of the malic, citric, and ascorbic acid contents, in addition to on the antioxidant activity and total phenolic content. Ayyappan et al. [160] assessed wheat cookies fortified with XOS at 5, 10, and 15% to explore their physicochemical characteristics, nutritional improvement, and storage stability. XOSs could enhance the functional value of the new product, yielding a 14% increase in the crude fiber and a 35% increase in the total dietary fiber compared the to control samples. Cookies containing 5% XOS retained a physicochemical stability similar to the control over 21 days at 25 °C, whereas high concentrations (10–15%) stimulated more marked changes in moisture distribution, giving rise to a softer texture and decreased the structural integrity. From a sensory perspective, the 5% XOS revealed the most required stability between enhanced nutritional value and acceptable texture, color, and overall consumer acceptance. In opposition, high XOS concentrations negatively affected the gas retention during baking, producing darker color and lower sensory acceptance. Wu and Lin [161] added several levels of XOS, trehalose, sucrose and sorbitol as cryoprotectant agents into meat batters. These authors confirmed that XOSs display a better cryoprotective effect than sucrose, trehalose, and sorbitol while evading extra caloric intake. Through frozen storage, Zhang et al. [33] verified that XOSs create hydrogen bonds with myosin, changing water molecules around the protein and thus modifying the damaging effects of ice crystal creation on myofibrillar proteins. Furthermore, XOSs interrelate with myosin to decrease chain elasticity and conformational variations, eventually improving the myofibrillar proteins’ stability.

### 7.3. Isomaltooligosaccharides (IMOs)

IMOs, employed as purposeful elements in the food industry, provide positive aspects like their high stability at a raised pH and temperatures, and their low viscosity and water activity, as well as their ability to fight crystallization, retain mild sweetness, and exhibit prebiotic activity. Lee et al. [162] demonstrated the high potential of IMOs as functional sucrose substitutes in sponge cakes, permitting a 25–100% replacement. Growing IMO syrup levels improved the batter’s viscosity and the cake volume, while decreasing specific gravity and the cake’s hardness. Sensory assessment exposed an enhancement in tenderness and overall acceptability, with cakes being less sweet as sucrose replacement was amplified. Over 3 days at 25 °C, a microbiological study revealed that the total plate counts surpassed 10^5^ colony forming units (CFU)/g, while refrigerated storage at 5 °C preserved counts below 10^3^ CFU/g for 7 days. Furthermore, the moisture content, water activity, color attributes, and IMO levels remained stable during storage, showcasing the industrial and storage appropriateness of IMOs in sponge cake formulations.

### 7.4. Galactooligosaccharides (GOSs)

GOSs present high solubility and high thermal stability covering a wide pH range, while displaying low caloric content and a sweetening power of about 35% that of sucrose [163]. GOSs are primarily utilized in infant milk preparations and infant foods. However, because of their physicochemical properties, they are also used in an extensive diversity of foods like beverages, mealtime substitutes, fermented milks, milk drinks, and confectionery products.

Arya and Shakya [164] examined GOS incorporation in functional multigrain beverages. The preparations had advanced dietary fiber and soluble solids levels, enhanced sensory acceptability, and unaffected color features. Throughout 10 days of storage, prebiotic-enriched beverages exhibited condensed phase separation, with GOSs providing the greatest stability. The established beverages were categorized by low caloric and glycemic values, respectable prebiotic potential, and high stability.

Balthazar et al. [165] examined the properties of GOS supplementation (1.5 and 3%) on the physicochemical, optical, and sensory properties of vanilla ice cream. GOS incorporation could improve the firmness of the product, reduce the melting rate, enhance stability, and increase overrun values. At 3% (*w*/*w*) GOS, the ice cream presented similar sweetness, flavor, and texture, and sensory characteristics compared to conventional prebiotics. Throughout yogurt production and storage, GOS at 2% leads to the development of functional traits via an enhancement in the viability of lactic acid bacteria strains, a shorter fermentation time, a higher acetic acid production, and an increase in proteolysis [166]. Prasad et al. [166] examined GOSs at 2% (*w*/*v*) in low-fat yogurt and found an enhancement in its fermentation performance and functional properties. The GOS addition stimulated the *Lactobacillus delbrueckii* subsp. bulgaricus growth, resulting in a briefer fermentation time. In addition, GOS-enriched yogurt displayed enhanced proteolytic activity and a higher production of organic acids, including lactic and acetic acids, compared with the control, particularly during the first 14 days of storage at 4 °C. Additionally, no significant changes in firmness were detected among the formulations, signifying that the texture was conserved. Overall, GOSs at 2% improve the starter viability, accelerate fermentation, and enhance bio-chemical activity without altering textural properties. Costa et al. [154] highlighted the potential of GOSs as a prebiotic ingredient in yogurt manufacturing. GOSs influenced the quality of Greek yogurt positively by enhancing its rheological characteristics, leading to more reliable, elastic, viscous, and safer products. In addition, GOS supplementation improved the volatile compound profile through the development of compounds linked to sweet and buttery flavors.

**Figure 1:**
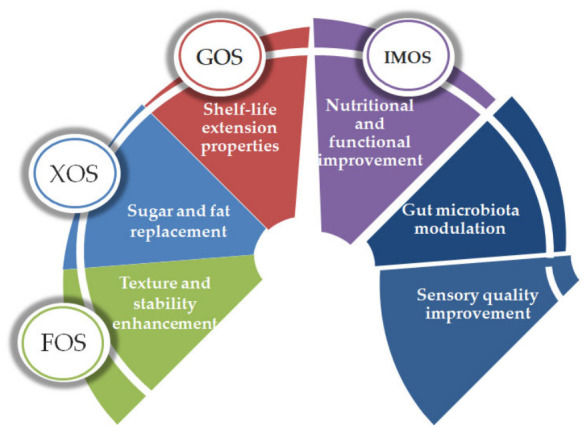
Main prebiotic oligosaccharides and their applications in the food industry.

In meat product application, Shin et al. [167] studied the effect of prebiotic oligosaccharides on the formation of heterocyclic aromatic amines (HAAs) in pan-fried beef patties, signifying that GOSs can successfully reduce the production of these toxic compounds. The authors stated that the addition of GOSs at 1.5% (*w*/*w*) into cooked beef patties decreased total HAA formation by 50%, attended by a 55% reduction in the overall mutagenicity. Fernandes et al. [168] detected the whey protein properties of biofilms with the addition of both GOSs/XOSs, recognizing exciting variations in functional and technological characteristics that could enhance value to whey protein film biopolymers, potentially applicable in meat engineering. Figure 1 summarizes the major prebiotic oligosaccharides (GOSs, FOSs, XOSs, and IMOs) employed in the food industry.

## 8. Toxic Chemical Carryover and Prebiotic Food Safety

During food processing, certain steps can involuntarily generate or introduce toxic compounds that may persist in the final food product if not properly controlled. High-temperature processes employed in food production, viz. extrusion, roasting, drying, or baking, can support the formation of process-induced toxicants. These contaminants include polycyclic aromatic hydrocarbons (PAHs), hydroxymethylfurfural (HMF), furans, acrylamide, nitrosamines, and advanced glycation end products (AGEs) [169]. For example, in lignocellulosic biomass valorization processes for oligosaccharide production, several undesired compounds can co-occur with the target fractions and pose contamination risks. Phenolic compounds derived from lignin, as well as sugar degradation products such HMF and furfural, are usually produced during acid or thermal pretreatment steps. If downstream purification steps (e.g., adsorption, membrane filtration, or extraction) are not efficient, these molecules may consequently persevere in oligosaccharide-rich fractions, particularly XOSs [169]. In prebiotic production, particularly from the agro-industrial by-products abundant in fibers and oligosaccharides, extra dangers may ascend from pesticide residues, mycotoxins, heavy metals, or contaminants that formerly existed in the raw material if pretreatment and obtaining are insufficient [170]. In addition, residual solvents used for extraction, like hexane, can also persist in ingredients and increase toxicological anxieties, which is why greener replacements like membrane filtration technologies are progressively preferred [170]. Furthermore, throughout storage and transport, contaminants may migrate from packaging materials or handling equipment into prebiotic preparations [171]. In this way, guaranteeing the safety of prebiotic ingredients requires a protective and combined approach, uniting careful raw material selection, and optimized processing conditions, contaminant tracking, Hazard Analysis and Critical Control Points (HACCP) application, and rigorous toxicological assessment, such as Food and Drug Administration (FDA) or European Food Safety Authority (EFSA) guidelines [172]. Overall, while prebiotic ingredients can be securely integrated into foods, preserving their protection is determined by regulatory contaminant formation and carryover through the whole production chain.

To guarantee the industrial security and governing agreement of enzymatically produced prebiotic ingredients, it is necessary to assimilate targeted plans that aim to diminish the formation and carryover of toxic compounds through the entire processing chain, including upstream processing, fermentation, and downstream purification. Process-induced contaminants (PICs), viz. acrylamide, heterocyclic amines, and HMF, can be diminished by working under mild enzymatic conditions, restraining thermal severity, and controlling precursor levels such as that of free asparagine and reducing sugars. In addition, targeted biocatalytic treatments by amylases, glucose oxidase, asparaginase, and proteases could reduce precursor levels before the thermal exposure.

Throughout fermentation, toxic metabolite development can be governed by starter culture selection, pH optimization, temperature, and fermentation period, thus limiting biogenic amines and linked compounds. In lignocellulosic processes, pretreatment-derived inhibitors such as phenolics, HMF and furfural, need effective elimination using activated carbon adsorption, membrane filtration, and advanced selective purification approaches accompanied by pH adjustment [169].

Assimilating these mitigation measures within an HACCP-based risk management framework, with critical control points at thermal, fermentation, and purification stages, confirms the dependable monitoring and qualification of chemical hazards, supporting both product safety assurance and industrial scalability [172].

## 9. Integration of Emerging Technologies in Prebiotic Oligosaccharides in Food Development

Owing to their nutraceutical properties and their sweet taste comparable to sucrose, prebiotics such as FOSs have been given particular consideration [20]. FOSs have been combined in numerous food products because they are easier to incorporate into some foods than other probiotics [173,174].

Filho et al. [175] assessed whether three non-thermal technologies are appropriate for FOS processing concerning its stability after processing. These authors stated that ultrasound and HPP have not impacted on FOS levels (<2.0%) nor on the polymerization degree of FOSs (<3.3%). The application of ultrasound before HPP could result in FOS hydrolysis since cavitation in aqueous solution produces free radicals that may possibly degrade FOS molecules.

Numerous studies have described the thermal processing of FOSs [176,177]. In order to compare shelf-life stability and the temperature and of arabinoxylooligosaccharides (AXOS), XOS, and FOS preparations, Courtin et al. [178] studied the impact of pH stability and heat (at 100 and 121 °C). It was reported that the FOS hydrolysis was augmented with increasing temperature. For all three preparations tested, at pH 11.0, heat stability exposed the decomposition of these prebiotics (at 400 and 800 Da for XOSs and FOSs, respectively. At pH 2.0 and 3.0, the hydrolysis of oligosaccharide relations took place, with FOSs being the most acid-sensitive components. The fructose furanosyl residues in FOSs are more disposed to acid hydrolysis than the pyranosyl units [25]. In addition, it should be noted that β-linkages, including xylan, are much more stable than are α-linkages, which link arabinose substituents to the xylan backbone [179]. The impact of heating (75–90 °C) and the polymerization degree (DP) on Sc-FOS stability in sodium citrate buffer and orange and tomato juices in acidic pH (pH 3.5) environments was presented by Vega & Zuniga-Hansen [180]. Principally, these authors stated that, under all of the tested conditions, pentasaccharides were more stable to heat treatment than trisaccharides. Additionally, the sc-FOSs were more stable in orange juice, succeeded by tomato juice and citrate buffer. In addition to pH and temperature, features like the DP, food matrix, and processing conditions (pasteurization) impact sc-FOS stability. In this study, the incessant thermal processing simulation for each of the corresponding processes at 90 °C exposed that the % of sc-FOS retention is more than 95%. Biochemically, the lower heat and low pH resistance of sc-FOSs compared with other oligosaccharides has been explained by the partial hydrolysis of the β(2 → 1) linkages between fructose units [181].

Gomes et al. [182] evaluate ultrasound processing and HPP effects on the bioactive compounds and quality of prebiotic cranberry juice. Chemical analyses were conducted to detect and quantify anthocyanins, in addition to assessing the FOSs, organic acids, antioxidant capacity, instrumental color, soluble solids, and pH. By using Ultra-Performance Liquid Chromatography—Quadrupole Time-of-Flight Mass Spectrometry (UPLC-qTOF-MS), seven anthocyanins were. Both non-thermal treatments effectively preserved FOS content, preserving the prebiotic functionality despite slight depolymerization. Organic acids showed high retention (>90%), while insignificant variations were perceived in relation to the soluble solids, color and pH. When managed by both technologies (US and HPP), anthocyanin content was augmented until 24%.

Almeida et al. [123] evaluated the impact of cold plasma (70 kV, 15–60 s) and HPP (450 MPa, 5 min) on the prebiotic orange juice enriched with 7 g/100 g of FOS. Chemical analyses, including of the FOS profile, organic acids, color, and vitamin C, showed that both treatments efficiently preserved the overall quality of the juice. Although HPP provoked the maximum changes in oligosaccharides, the prebiotic performance stayed acceptable. As such, no noticeable color changes were observed (ΔE < 3), while vitamin C and citric acid contents were well preserved or even slightly enhanced after processing. According to Silva et al. [183], the supercritical CO_2_ processing of inulin-enriched apple juice established clear advantages over conventional thermal treatment. The growing pressure levels (10–20 MPa at 35 °C for 10 min) resulted in a linear reduction in particle size, enhancing dispersion stability without destruction, which could affect the key physicochemical attributes. A treatment of 95 °C/1 min could degenerate inulin into short-chain FOSs. On the other hand, supercritical CO_2_ conserved the integrity of the inulin structure and maintained the organic acids, natural sugars—glucose, fructose, sucrose, and sorbitol—and phenolic compounds. Significantly, antioxidant activity (DPPH and TEAC) remained untouched, ensuring the preservation of functional quality. Later, Silva et al. [184] valued the chemical stability of XOSs in orange juice subjected to HPP under 30 °C and thermal 100 °C at pressures reaching between 100 and 600 MPa. Under intense mechanical and thermal conditions, XOSs preserved their chemical integrity, approving their vigor during processing. HPP at 100 °C endorsed sucrose hydrolysis into glucose and fructose, while retaining their key physicochemical properties like organic acids (malic and citric), soluble solids, pH, ζ-potential, total phenolic content, and antioxidant activity.

Guimarães et al. [185] examined the nutritional profile and volatile compounds existing in inulin soursop whey beverage in the existence of high-intensity ultrasound (HIUS) at 200–600 W. Generally, HIUS resulted in important changes in the beverage’s quality, with both valuable and opposing impacts. In this study, the content of phenolic compounds increased after HSTS (16%, *p* < 0.05) and US 200, US 400 and US 600 (10, 14 and 18% respectively, *p* < 0.05). HIUS at 400 and 600 W presented phenolic compound levels comparable to high-temperature short-time (HTST). The increase in phenolic levels with the rise in ultrasound power is attributed to the amplified acoustic cavitation and mechanical shear forces, which could disturb cell vacuoles and walls, simplifying the release of bound phenolics [186].

Ribeiro et al. [124] studied cold plasma application (0, 5, 10, and 15 min) to prebiotic whey-based beverages fortified with XOS at 1.5% (*w*/*v*). The effects on physicochemical properties, XOS stability, rheology, bioactive compounds, and sensory attributes were investigated. Compared to untreated samples, cold plasma could preserve XOS stability and maintain the sugar composition. In addition, apparent viscosity and consumer acceptance persisted generally unaffected; however, a reduction in color intensity and consistency (K = 4.31–42.21 mPa·s^n^, n = 0.57–0.95) was achieved. Furthermore, compared to pasteurized samples, treated beverages with cold plasma displayed inferior heat harm indicators, with reduced HMF concentration at 1.91–2.10 µmol/L, and a developed whey protein nitrogen index (WPNI: 6.1–6.7 µmol/L). Moreover, cold plasma improved the concentration of bioactive compounds. In this study, the antioxidant activity, the inhibitory effects against angiotensin-converting enzyme (ACE), and α-amylase and α-glucosidase activities were improved.

## 10. Mechanistic Prebiotic Behavior Under Thermal and Non-Thermal Processing

Non-thermal technologies are gaining increasing attention in relation to prebiotic processing. On the other hand, the efficiency of prebiotics is strongly predisposed by their molecular structure which included DP, glycosidic linkages, branching and MW. In addition, thoughtful how non-thermal processing affects these structures is indispensable for optimizing fermentability, gut microbiota modulation, and overall biofunctional performance.

### 10.1. Advanced Non-Thermal Processing of Prebiotics

Non-thermal technologies like high-pressure processing (HPP), high-intensity ultrasound (HIUS), and cold plasma (CP) have gained increasing attention in prebiotic food processing due to their ability to enhance microbial safety and product stability. Unlike conventional thermal treatments, which often provoke the degradation of FOSs, inulin, and related oligosaccharides, these technologies keep the structural integrity of prebiotic carbohydrates. Their effects are primarily based on physical or localized chemical mechanisms that exert minimal impact on the covalent backbone of these bioactive molecules [140]. This merit is predominantly significant since the health benefits of prebiotics are largely contingent on keeping their chain length, DP, and resistance to enzymatic digestion. At the molecular level, HPP acts through the use of unchanging isostatic pressure that mostly interrupts non-covalent interactions like hydrogen bonds and hydrophobic associations. while glycosidic bonds remain largely untouched under typical food-processing conditions. As a result, FOS levels and DP varied by less than 3.3% even after treatment at 450 MPa for 5 min; however, thermal processing provoked marked hydrolysis and loss of functionality [140]. In prebiotic-enriched orange juice, HPP prompts only slight FOS breakdown while maintaining color and general sensory quality more efficiently than heat treatments [148]. In more complex food systems, pressure-induced protein deterioration and polysaccharide gelation may also generate protective microstructures that substantially protect prebiotic compounds from further fragmentation [140,150]. HIUS operates through acoustic cavitation, where microscopic bubbles rapidly form and collapse, generating localized zones of extreme pressure, shears forces, high temperature, and transient free radicals [187]. HIUS leads to the remarkable stability of many prebiotic carbohydrates throughout processing [11]. In inulin-enriched whey beverages, HIUS mostly touches protein organization, endorsing the whey protein loss of native structure and interactions with inulin and gellan that enhance gel structure, decrease particle size, and improve the colloidal stability deprived of detectable inulin degradation [150]. Likewise, FOSs processed with ultrasound (600–1200 W/L for 5 min) display no alteration in DP, demonstrating that cavitation-generated radicals are inadequate to lengthily hydrolyze glycosidic bonds below measured conditions [140]. Combined ultrasound–HPP treatments in FOS-enriched cranberry juice also keep prebiotic carbohydrates while improving anthocyanin extraction through mass transfer and cell disturbance rather than carbohydrate interruption [147]. Cold atmospheric plasma works through reactive oxygen and nitrogen species (RONS), UV photons, charged particles, and electric fields that can change biomolecules through oxidation and structural reorganization [188]. Nonetheless, prebiotic carbohydrates perform moderately resilient to plasma-induced decomposition [140]. In prebiotic orange juice, plasma treatment preserved functionality and color while growing quantifiable citric and ascorbic acid levels, likely due to the improved release from the food matrix [148]. Overall, lipids and proteins are commonly more sensitive to plasma-induced oxidation, whereas prebiotic carbohydrates show better structural stability, predominantly when added in complex food matrices. Overall, whereas thermal processing could promote oligosaccharide hydrolysis and chain fragmentation, HPP, HIUS, and CP mainly target non-covalent interactions, microbial cells, or matrix components before significantly disturbing carbohydrate covalent bonds. This discriminating action allows prebiotics to retain their structural reliability, DP, and functional characteristics while concurrently enhancing the product safety and food quality.

Because of their aptitude for reserving bioactivity and improving process sustainability, HPP, CP, and HIUS are well exploited for prebiotic carbohydrate processing. Comparative evidence suggests that FOSs persist extremely constantly under these treatments, with minimal changes in their concentration and DP, approving their suitability for industrial applications. HPP supports uniform volumetric treatment and robust product quality retention but is inhibited by high capital costs and batch operation. Regarding CP, this treatment is operative for bioactive preservation surface and decontamination; however, its imperfect diffusion depth, non-uniform release, and scale-up encounters confine its industrial deployment. HIUS improves the extraction, dispersion, and process intensification, yet suffers from varied energy flux distribution and partial reactor scalability. In prebiotic systems, while each technology proposed different advantages, their industrial implementation faces economic and technical barriers. Therefore, hybrid and combined processing approaches joining non-thermal technologies with enzymatic and conservative approaches seem most talented for attaining well-organized, scalable, and sustainable prebiotic food production.

### 10.2. Molecular Structure and Prebiotic Functionality

**Figure 2:**
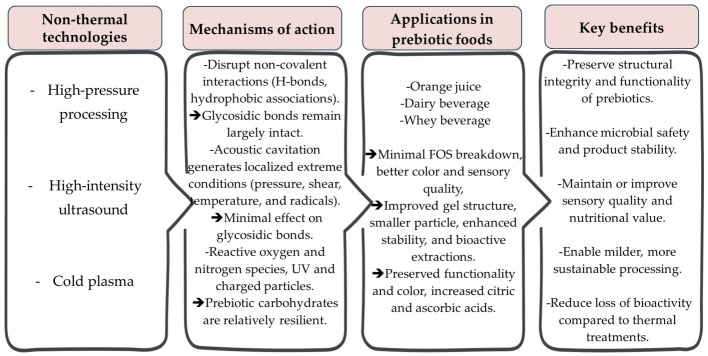
Applications and mechanisms of non-thermal technologies in prebiotic food Processing.

The techno-functional performance of prebiotics generated from agro-industrial wastes is substantially impacted by their molecular structure. This is evident when they are treated by means of developing non-thermal technologies like CP, HPP and HIUS. These technologies dynamically adapt carbohydrate architecture at the molecular/supramolecular stages, counting chain length, dividing degree, crystallinity, molecular weight distribution, and surface features. Such structural variations impact the techno-functional possessions of prebiotics, encompassing solubility, water-holding capacity, viscosity, gelation behavior, stability, and relations within food matrices. Based on the treatment intensity and substrate profile, cold plasma treatment can elicit both the depolymerization and cross-linking of carbohydrate chains [87]. In prebiotic-rich polysaccharides and starch-derived oligosaccharides, plasma produced reactive species and changed molecular associations deprived of widespread heat-activated decomposition [123,124]. For example, plasma provoked cross-linking and could intensify the water-binding capacity, swelling power, and viscosity by generating more planned and consistent carbohydrate nets, while partial depolymerization can augment solubility, paste clarity and stability by reducing chain length and preventing retrogradation [125]. HPP principally modifies non-covalent interactions inside carbohydrate assemblies while keeping the glycosidic bonds that are indispensable for prebiotic functionality. Under slight temperatures, HPP adjusts the supramolecular organization of polysaccharides and oligosaccharides, decreasing crystallinity and aiding gelation or viscosity expansion. This fact could be explained structurally by perturbing hydrogen bond systems and aiding water penetration [126]. In prebiotic systems, these structural reorganizations can expand texture, water retention, and suspension stability without expressively demeaning sensitive oligosaccharide chains [128]. By means of acoustic cavitation, HIUS touches prebiotic carbohydrates, where collapsing microbubbles produce powerful restricted shear forces and turbulence [128]. These properties interrupt lignocellulosic materials, recover mass transfer, and simplify the extraction of prebiotic polysaccharides such as β-glucans, hemicelluloses, mannans, and fructans from agricultural by-products [128]. At the same time, HIUS-encouraged depolymerization and granule surface alteration alters molecular weight and carbohydrate organization. This fact has a direct impact on viscosity, swelling behavior, solubility, and gel formation. In liquid systems, sensible molecular weight might expand prebiotic dispersibility and functionality, while extreme depolymerization could harmfully disturb viscosity and structural stability. HIUS treatments have also been related to modified rheological behavior and enhanced hydration due to the loosening of carbohydrate networks and increased surface accessibility [129]. General, under non-thermal processing, prebiotics’ functionality is powerfully impacted by how these skills redesign the carbohydrate structure. Changes in chain length, branching, crystallinity, and surface chemistry could control hydration, viscosity, gelation, stability, and food matrix relations. In this way, launching structure/process/function associations suggests a mechanistic framework for producing prebiotic ingredients from agro-food wastes and enhancing their performance in sustainable functional food applications. Figure 2 highlights a comparative overview of high-pressure processing, high-intensity ultrasound, and cold plasma, emphasizing their mechanisms of action, food-related applications, and advantages in preserving prebiotic functionality and product quality.

## 11. Integrated Assessment of Enzyme and Energy Burdens in Waste to Prebiotic Conversion

### 11.1. Enzyme Costs in Waste to Prebiotic Processes

In enzymatic prebiotic production, the main cost is enzymes and their dosage. A higher enzyme demand or the need for intensive pretreatment increases the overall process cost and reduces the economic viability of converting lignocellulosic biomass into prebiotics. In many cases, enzyme systems strongly affect both the initial investment cost and final product value. For instance, in FOS production, free enzymes may initially perform cost-effectively, but variations in enzyme market prices can speedily move the balance toward immobilized systems or enzyme recycling policies [1].

In addition, the usage of expensive commercial enzymes can reduce the keenness of biorefinery conceptions, mostly when enzymes cannot be improved or recycled. In contrast, on-site enzyme production can enhance overall feasibility by decreasing supply chain dependence and cost instability. Correspondingly, enzyme immobilization and recovery systems can lower working costs by decreasing enzyme consumption and extending the catalyst lifetime over numerous cycles. In addition to direct costs, enzyme manufacture generates a substantial environmental and energy footprint. In some circumstances, industrial enzyme production produces significant waste and requires significant amounts of energy, tightfitting the often ignored impacts of production, purification, and stabilization steps. At the industrial scale, commercial enzymes can become major contributors to operative costs and overall energy consumption. The optimization of reaction parameters (temperature, pH, and enzyme) can augment conversion effectiveness while reducing enzyme practice. In addition, immobilized enzyme systems and membrane-assisted reactors can recover enzyme recycling and abridge downstream processing. Lastly, integrated biorefinery methods that co-produce multiple value-added products or energy carriers can support the balance of enzyme and energy costs, thus improving the overall process’s productivity and sustainability.

**Figure 3:**
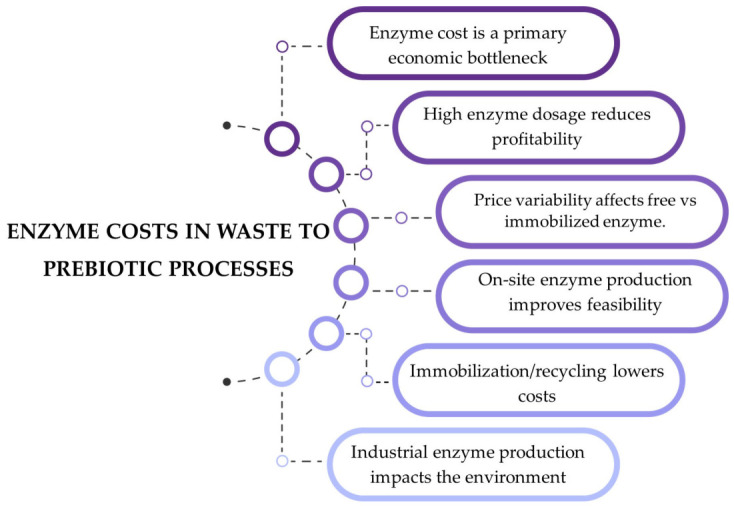
Economic challenges of enzymatic waste to prebiotic bioprocesses.

Figure 3 illustrates the main cost drivers and the economic feasibility of waste-to-prebiotic production, as well as enzyme expenses, dosage requirements, recycling strategies, and environmental impacts.

### 11.2. Framework for Quantitative Modeling of Prebiotic Fermentation

In prebiotic investigations, mass balance frameworks are attracting increasing attention since they deliver a mechanistic way to realize how prebiotic substrates are engineered in biological and industrial environments. Recent studies have removed the barrier to predictive and quantitative methods that track the movement of carbon, electrons, and metabolites during microbial fermentation. Flux balance analysis (FBA) and community-scale metabolic modeling are recent approaches which are extensively employed to clarify how precise prebiotics [189], including FOSs, GOSs and XOSs, are digested by intestinal microorganisms and rehabilitated into physiologically significant metabolites such as acetate, propionate, butyrate, hydrogen, and CO_2_ [42]. These models establish direct relations among the substrate structure, microbial metabolism, and functional health outcomes enabled by measuring the balance between microbial biomass synthesis and helpful metabolite generation from prebiotic carbon [190].

An important strength of these frameworks is their aptitude to characterize the gut microbiota as a dynamic metabolic ecosystem. In the colon, prebiotic fermentation comprises wide cross-feeding interactions where one microbial group partly reduces complex carbohydrates into smaller intermediates, which are then used by other microorganisms [191]. For example, *Bifidobacteria* may originally hydrolyze oligosaccharides into lactate and acetate, which are then expended by butyrate-producing bacteria to engender extra health-promoting metabolites [42].

**Figure 4:**
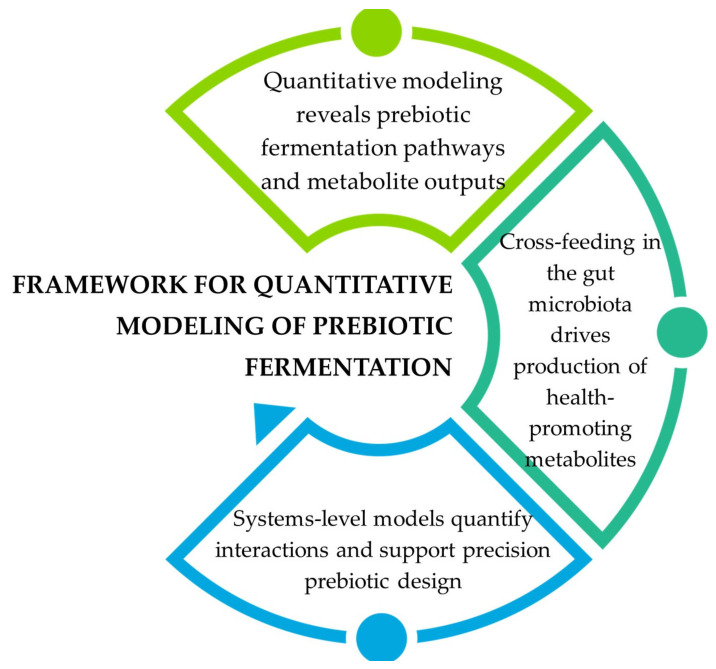
Framework for quantitative modeling of prebiotic fermentation.

At the microbiota level, mass balance and metabolic network models facilitate the quantification of the synergetic pathways and elucidate how prebiotics effect microbial diversity, stability, and metabolite synthesis [192]. This systems-based insight is progressively supporting the progress of “precision prebiotics”, precisely intended to encourage targeted microbial functions or metabolic outputs [193]. Figure 4 explains microbial interactions, cross-feeding mechanisms, and metabolite formation during prebiotic fermentation, supporting predictive modeling and precision prebiotic design.

### 11.3. Life Cycle and Techno-Economic Assessment of Prebiotic Production

**Figure 5:**
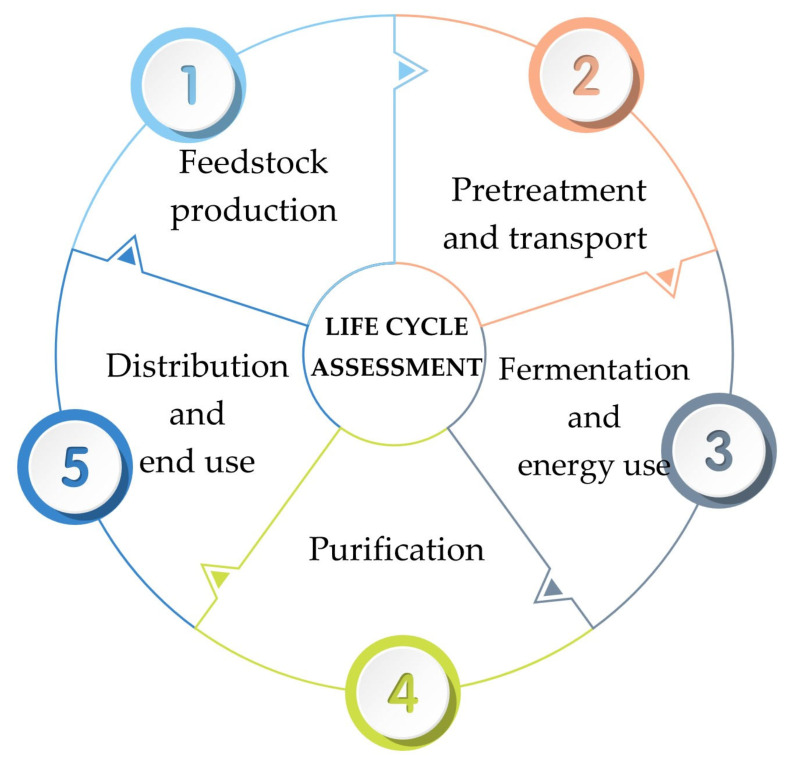
Life cycle assessment (LCA) of prebiotic production from waste biomass.

From a sustainability viewpoint, life cycle assessment (LCA) and techno-economic analysis (TEA) are progressively useful for determining the environmental and economic feasibility of prebiotic production systems [194,195]. Figure 5 outlines the main stages considered in the LCA of prebiotic production, including feedstock generation, transportation, processing, purification, distribution, and end use.

The largest environmental and economic hotspots are linked to feedstock production, fermentation energy necessities, and solvent- or membrane-based purification processes. This is especially pertinent for prebiotics obtained via waste bioconversion, where transport, pretreatment, and purification can significantly affect the overall environmental footmark. Nonetheless, prebiotic-specific LCA and TEA investigations continue to be comparatively incomplete, and many investigations still trust assumptions borrowed from broader bio-based production systems, complicating inter-study assessments [195].

**Figure 6:**
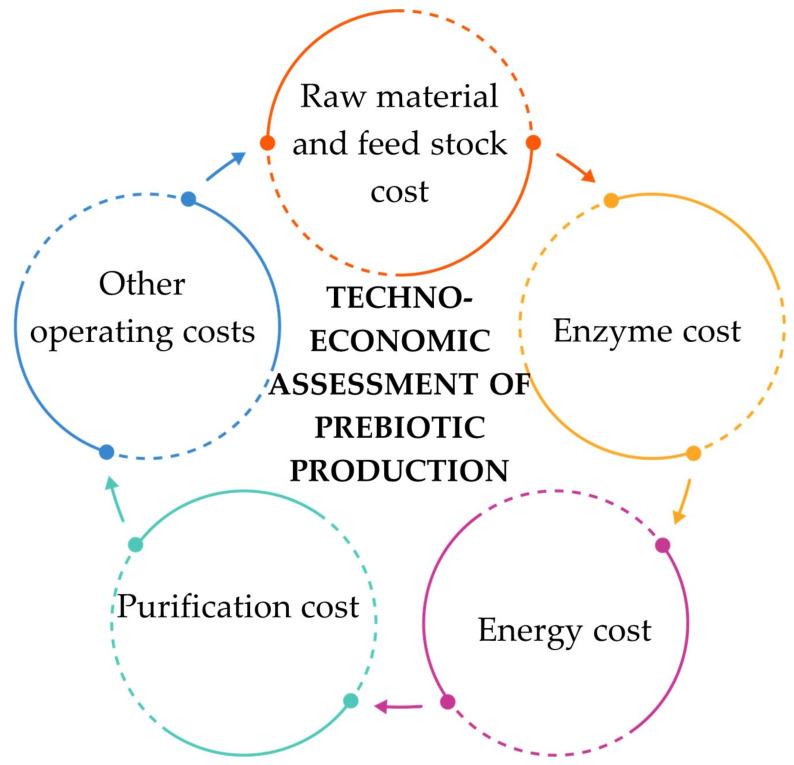
Techno-economic assessment (TEA) of waste-derived prebiotic manufacturing.

Figure 6 describes the key economic parameters considered in the TEA of prebiotic production, including operational and feedstock-, enzyme-, energy-, and purification-relate costs that determine overall process feasibility and scalability.

Future development in prebiotic research will need combined frameworks that concurrently associate microbial-scale carbon and electron balances, full industrial procedure mass accounting, and explicit environmental and economic valuations. Such integrated methods will be indispensable for scheming scalable, resource-efficient, and maintainable prebiotic production systems suitable for supporting the expanding demand for functional foods and microbiome-oriented nutrition.

## 12. Conclusions

From agro-industrial by-products, prebiotic oligosaccharide production has been demonstrated to be a real strategy of the circular economy. Enzymatic processes’ integration with waste valorization can meaningfully improve prebiotic oligosaccharides’ production efficiency. Furthermore, developments in relation to biotechnological production, chiefly in terms of enzymatic synthesis and fermentation using microbial approaches, have evidently enhanced the yield, structural specificity, and scalability of prebiotic oligosaccharide engineering. These revolutions are converting prebiotic oligosaccharide production and related final food products with distinct physicochemical and biological properties. In addition, the incorporation of prebiotic oligosaccharides into the food matrix not only improves dietary patterns but also triggers technological, physicochemical, and sensory variations. Furthermore, prebiotic oligosaccharides can substitute for ingredients allied with undesirable health consequences.

To completely exploit prebiotic oligosaccharides’ potential and solve their marketable value, new innovative research directions should address numerous important domains. In this context, cumulative novelties could underlie the elucidation of structure–function relationships, which aid in connecting specific prebiotic oligosaccharides’ physicochemical properties with biological and health impacts. These innovations have located prebiotic oligosaccharides as key ingredients for conventional food systems and next-generation functional and tailored nutrition. In addition, prebiotic oligosaccharides’ application as preservative additives in food products still necessitates supplementary examination, as their potential as shelf-life agents still remains unsatisfactorily illuminated. In this context, additional studies are required to validate their application as functional ingredients in novel food preparations with satisfactory sensory features.

## Figures and Tables

**Table 1:** Summary of FOS production systems.

Source	Microorganism	Enzyme	Production Conditions	Main Results	References
Aguamiel from Agave salmiana	*Aspergillus oryzae* DIA-MF	FTase	SSF; inoculum 10 × 10^7^ spores/mL; 72 h, 28 °C, pH 5.0	FOS yield: 20.30 g/L; Productivity: 0.84 g FOS/L·h.	[31]
Agricultural waste materials	*A. flavus* NFCCI 2364	FTase	SSF; inoculum 10 × 10^8^ spores/mL; 96 h; pH: 5.0/28 °C	FTase production: 423.18 U/g dry substrate.	[30]
Coffee silverskin	*A. japonicus* ATCC 20236	FTase	SSF; inoculum 2 × 10^6^ spores/g, 48 h, 28 °C, pH 5.0	FOS production: 128.7 g/L; FTase activity: 71.3 U/mL, FOS productivity: 8.05 g/L⋅h	[32]
Coffee silverskin	*A. japonicus* ATCC 20236	FTase	SSF; inoculum 2 × 10^6^ spores/g, 48 h, 28 °C, pH 5.0	FOS production: 208.8 g/L; FTase activity: 64.12 U/mL productivity: 4.0 U/mL h	[35]
Agro-industrial residues	*A. niveus*	β-FTase	SSF; inoculum 10^5^ spores/mL spores/mL; 72 h, 40 °C	β-FTase half-life = 53 min/60 °C, enhanced by Cu^2+^, Mn^2+^ and Mg^2+^	[36]
Sugar syrup and molasses	*A. aculeatus*	FTase	Pectinex Ultra SP-L/Sepabeads EC-EP5, 24 h.	FOS production: 235 mg/mL (≈49% *w*/*w*) after 65 h using molasses.	[37]
Date By-products	*A. awamori* NBRC4033	β-FTase	Immobilization on chitosan by covalent binding	FOS: 123 g/L; Sucrose conversion: 53.26%; Productivity: 18.5 g/h/100 g substrate	[34]
Cane Molasses	*Aureobasidium melanogenum*	β-FTase	375 U/g sugar, 50 °C/pH 4.5/100 rpm.	2100 U/mL β-FTase; 350 g/L cane molasses sugar; 4 h; FOS yield: 0.58 g/g substrate.	[33]
Molasses	*A. japonicus*-FCL 119T and *A.niger* ATCC 20611	β-FTase	Sucrose (3–25%) or cane molasses (3.5–17.5%); peptone (2–5%).	FOS yield: ~60% *w*/*w* of total sugars	[38]
Wheat bran	*Fusarium graminearum* HB0810	β-FTase	SSF; 10^6^ spores mL; 7 days, 30 °C, pH 5.0	8× purification (14% recovery); 94/66 kDa; 55–60 °C, pH 4.5; stable at 30–50 °C,	[39]
Banana leaf (BL), GOC: groundnut oil cake	*Saccharomyces cerevisiae* GVT263	β-FTase	BL/GOC as C/N sources; MnSO_4_, inoculum	Optimized FFase production: 3587 U/mL (9-fold increase) in 48 h	[40]
Cassava wastes	*Rhizopus stolonifer* LAU 07	FTase	24 h, 20%, pH 5.5, 30 °C, 100 rpm, 120 h.	FFase: 45.7 U/mL; FOS yield: 34%; biomass: 8.8 (24 h), 10.0 (72 h), 5.6 g/L (120 h).	[41]

**Table 2:** Overview of XOS production processes.

Source	Microorganism	Enzyme	Production Conditions	Main Results	References
Agro-industrial biomass	Commercial enzymes	Endo-xylanase, α-L-arabinofuranosidase, feruloyl esterase.	Enzymatic hydrolysis	High XOS yield, stability and antioxidant potential	[64]
*Miscanthus*	Not specified	Endo-xylanases NS22083 and NS22002	Steam explosion (200 °C; 15 bar; 10 min)	50% xylan → XOS; 70% glucan hydrolysis; 8–9× glucose	[72]
Not specified	*Pichia stipitis*	Xylanase	Fermentation with enzymatic hydrolysis	SSF xylanase: 5536 U/g; 31.6 kDa; Kcat/Km 301 mL/s·mg	[68]
Barley malt residue	Not specified	Xylanase	Microwave-assisted enzymatic hydrolysis (MAEH)	208.05 mg/g XOS; RSM 1235 W, 6 min, 89.12 U/g xylanase; *S. cerevisiae* > conventional methods.	[73]
Grape stalks	Not specified	Endo-β-1,4-xylanase M3 (*T. longibrachiatum*) + deacetylase.	Controlled enzymatic hydrolysis	Controlled DP XOS production; mainly DP2–DP4 (60–96%), with ~11% DP7–DP10.	[74]
Beechwood	*Aureobasidium pullulans* CCT 1261	Crude xylanase	T° (40–50 °C), Time (12–48 h), 6%, *w*/*v*), and pH (4–6)	XOS optimum: 180 rpm, 40 °C, 24 h; 10.1 mg/mL	[70]
Agricultural waste	*Aspergillus fumigatus* R1	Extracellular xylanase	Submerged fermentation: 96 h, 37 °C, 100 rpm;	Xylanase 152 IU/mL; XOS yield 1%; 60% strains utilized XOS	[75]
Sugarcane bagasse	Not specified	Xylanase	RSM-optimized hydrolysis (T°, time and enzyme (U/L)	Optimal XOS production: pH 4.75, 45 °C, 4 U/mL enzyme, 16 h	[71]
Green coconut and vegetable cocktail.	Not specified	Xylanase hydrolysis after chemical pretreatment (HP–AC and SH–SH)	Pretreatment: 80 °C, 4% NaClO (2 h) → 0.08% NaOH (55 °C, 1 h).	Xylopentose-dominated XOS profile (96.44% GC; 93.09% VC)	[76]
Brewers’ spent grain	*Trichoderma reesei*	Xylanase	3 days, pH 7.0, 30 °C and 20 g/L	-AXOS: 40 mg/g (xylose eq, BSG); DP 2–5; one-step fermentation	[77]
Sugarcane bagasse	*Bacillus stercoris* DWS1	Xylanase (β-xylosidase-free)	pH (7), T° 37–60 °C	Xylanase: 591 U/mL (~35 kDa), XOS: 400 mg/g, mainly xylobiose/xylotriose	[78]
Not specified	*Thermomyces dupontii* J22	Xylanase	5% C source; 3% N mix (yeast extract:tryptone:NH_4_H_2_PO_4_ = 3:1:1); pH 7.2; 47.5 °C	Xylanase: optimal activity at 80 °C, pH 6.0; XOS production optimal at 60 °C, pH 7.0: xylobiose/xylotriose (74.29%).	[79]
Not specified	*Pichia pastoris*	Recombinant xylanase	Heterologous expression	Antarctic GH10 xylanase (Xyl-L) = 5.10 U/mg; Km 3.5 mg/mL; Kcat 9.16/s (pH 7.5).	[47]
Agricultural residues	Not specified	Thermostable xylanase expressed and secreted by *Pichia* pastoris.	Bioprocess optimization	Recombinant tXyn2: 10.2 g/L (5 L bioreactor); saccharification 65.3% (corncob) and 77.5% (cottonseed hull)	[80]

**Table 3:** Representative cases of enzymatic production of isomaltooligosaccharides (IMOs) from agro-industrial wastes.

Source	Microorganism	Enzyme	Production Conditions	Main Results	References
Sweet potato starch	*Saccharomyces cerevisiae* Var. diastaticus BE 134	Transglycosylation enzymes	Simultaneous saccharification and transglycosylation	Spezyme Xtra: ~74% oligosaccharides; SST enzymes: IMOs up to 70 g/L.	[95]
Maltose	*Bacillus* subtilis AP-1	Cell-bound α-glucosidase with transglucosidase activity.	Direct fermentation; 50 g/L maltose; 36 h cultivation	IMOs (DP 2–14) via maltose fermentation; 37 g/L (72.7% yield); strong prebiotic activity, stable and non-toxic (≤5 mg/mL).	[98]
Potato peel starch	Recombinant *E. coli* producing *A. niger* enzyme	GH31 α-transglucosidase immobilized on silica-coated magnetic nanoparticles.	3 steps: liquefaction, saccharification, transglucosylation; optimum: 45 °C, pH 5.5, 6.9 U/g, 9 h	Enhanced transglucosylation: 6.9 U/g enzyme, 9 h, 45 °C, pH 5.5, yield = 70 g/L; IMOs produced with DP 2–10	[96]
Maltose	*Zalaria* sp. Him3 (yeast)	Secreted α-glucosidase (AGase)	280 g/L maltose; 30 °C; 0.25 U/mL AGase; 72 h	*Zalaria* sp. Him3: AGase 0.0165 U/mL; IMOs 138 g/L (49.5% yield); ~98% conversion	[99]
Maltodextrin	Recombinant *Thermoanaerobacter thermocopriae* TG	Novel transglucosidase (TtTG, GH15/CBM35)	Optimum pH 4.0; 60 °C; maltodextrin as main substrate	TtTG: 94 kDa; optimum pH 4.0, 60 °C; broad acceptor specificity for oligosaccharide synthesis.	[100]
Tapioca starch	Recombinant amylomaltase + *A. niger* transglucosidase	Amylomaltase (WT or Y101S) + transglucosidase	30% (*w*/*v*) soluble tapioca starch; 40 °C; 120 U amylomaltase + 6 U TG; 30–60 min	Optimal IMOs: 30% tapioca starch, 120 U amylomaltase + 6 U transglucosidase, 40 °C (30–60 min); α-1,4/α-1,6 linkages, DP ≤ 9	[101]
Wastes: Soluble starch	Commercial enzymes	Fungamyl 800L (saccharification) + α-glucosidase (transglucosylation)	Simultaneous saccharification–transglucosylation (SST); 12 h vs. 24 h CST	95 g/L (12 h) vs. 89.66 g/L (24 h, CST); 0.06 U Fungamyl + 1.0^5^ U α-glucosidase; scalable (PPW 92.17 g/L, broken rice 85.11 g/L).	[97]
Kimchi: vegetable matrix	*Leuconostoc citreum* KACC 91035	Dextransucrase (glycosyltransferase)	Fermentation at 10 °C; sucrose 58 mM (donor) + maltose 56 mM (acceptor)	Starter addition: IMO rate 15.05 → 22.04 mM/d; concentration 75.27 → 110.19 mM	[102]

## Data Availability

No new data were created or analyzed in this study. Data sharing is not applicable to this article.
